# Tumor-stroma type and tumor-stroma ratio predict neoadjuvant chemotherapy response in breast cancer

**DOI:** 10.1590/1806-9282.20241225

**Published:** 2025-03-31

**Authors:** Oğuzhan Okcu, Çiğdem Öztürk, Anıl Can Yalçın, Bayram Şen, Nazlıcan Yalçın, Ezgi Hacıhasanoğlu, Esra Aydın

**Affiliations:** 1 Recep Tayyip Erdoğan University, Faculty of Medicine, Department of Pathology – Rize, Türkiye.; 2 Recep Tayyip Erdoğan University Training and Research Hospital, Department of Pathology – Rize, Türkiye.; 3 Recep Tayyip Erdoğan University Training and Research Hospital, Department of Biochemistry – Rize, Türkiye.; 4 Yeditepe University, Faculty of Medicine, Department of Pathology – İstanbul, Türkiye.; 5 Recep Tayyip Erdoğan University Training and Research Hospital, Department of Oncology – Rize, Türkiye.

**Keywords:** Breast neoplasms, Tumor microenvironment, Neoadjuvant therapy, Stromal cells, Prognosis

## Abstract

**OBJECTIVE:**

Breast cancer is the most common cancer type among women. One of the most important parameters in the prognosis of patients is the response to neoadjuvant chemotherapy. The most important parameter for neoadjuvant chemotherapy success is appropriate patient selection. We investigated the effect of tumor-stroma type and tumor-stroma ratio on neoadjuvant chemotherapy response, using the Residual Cancer Burden scoring systems.

**METHODS:**

Patients diagnosed with breast carcinoma in core needle biopsy materials between 2010 and 2023 and whose neoadjuvant treatments and surgeries were performed in our institution were scanned from the database. A total of 158 patients who met the study criteria were included in the study.

**RESULTS:**

Tumor-stroma ratio and collagen-dominant tumor-stroma type were associated with neoadjuvant chemotherapy resistance, and tumor-stroma ratio was found to be an independent risk factor in treatment response. The probability of response to neoadjuvant chemotherapy treatment was higher in luminal molecular subtype breast cancer patients with low tumor stroma.

**CONCLUSION:**

An effective risk analysis for neoadjuvant chemotherapy treatment is not always possible with current clinicopathological parameters. Tumor-stroma ratio and tumor-stroma type seem useful in predicting neoadjuvant chemotherapy response as a reproducible practical marker and do not require additional cost and time.

## INTRODUCTION

Neoadjuvant chemotherapy (NAC) has been used increasingly in routine treatment in recent years in breast cancer (BC). The aim of NAC is to reduce tumor volume to prevent radical mastectomy and axillary lymph node dissection. Patients with complete response after NAC treatment showed better survival than patients with residual disease^1–3^.

The most important parameter for NAC success is appropriate patient selection. In routine practice, the molecular subtype is used to predict NAC response. Although the pathological response is better, especially in triple-negative (TN) and HER2 molecular subtype cases, the results may vary even within these groups^4^. Therefore, there is a need to investigate new parameters in addition to molecular subtypes in the prediction of NAC response.

Studies evaluating tumor stroma have reported that the tumor-stroma ratio (TSR)^5–10^ and tumor-stroma type (TST)^11^ are associated with overall survival (OS) and disease-free survival (DFS) in BC. Despite the consensus on the prognostic effect of tumor stroma on survival, studies from different centers are needed to determine its predictive value on NAC response.

In our study, we investigated the effect of TST [collagen dominant (C.D), fibromyxoid dominant (F.D), lymphocytic dominant (L.D)], and TSR along with various other pathological parameters on NAC response in invasive BC in core needle biopsy materials using the Residual Cancer Burden (RCB) scoring systems to find a parameter that predicts NAC response.

## METHODS

### Study design and case selection

Patients diagnosed with BC in core needle biopsy materials at our institution between 2010 and 2023 were scanned from the electronic database. During the selection process, patients who received NAC treatment and whose follow-up and surgical treatment were performed in our institution were identified. Patients lacking sufficient clinical data or who had distant metastases at the time of diagnosis were excluded from the study, and the remaining 158 patients were included in the study.

### Patient data

Clinicopathological data of the patients were obtained from the hospital database and pathology reports. ER (SP1, Ventana), PR (1E2, Ventana), HER2 [anti-HER-2/neu (4B5, Ventana)], and Ki-67 (30-9, Ventana) immunohistochemical stained preparations were reevaluated and rescored. Nuclear staining over 1% was considered positive for ER and PR, and complete membranous staining over 10% was considered positive for HER2^12^. A Ki-67 proliferation index of 14% and above was considered a high proliferation index^13^.

### Outcomes

DFS was defined as the time from the date of surgery to the radiological or pathological identification of metastases/recurrences or the date of the last follow-up. Overall survival was defined as the time from the date of the core needle biopsy to the date of the patient's last follow-up or the date of death.

### Tumor-stroma type, tumor-stroma ratio methodology, and residual cancer burden score

H&E-stained sections which were obtained from paraffin blocks of core needle biopsy and resection materials that were subjected to 10% formaldehyde fixation for 24–72 h were evaluated by two pathologists (OO and ÇÖ), blinded to the clinicopathological information of the patients. Pathologists evaluated using an Olympus BX-51 microscope equipped with a 22-mm ocular lens, and they independently classified TST and TSR. When disagreement occurred between two researchers, reevaluation was performed under a double-headed microscope.

**Figure 1:**
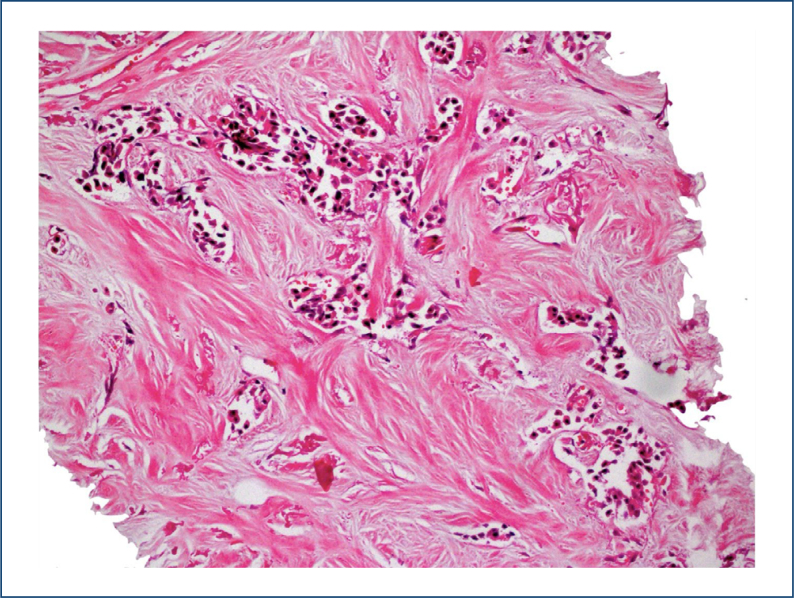
Breast carcinoma, high tumor-stroma ratio, collagen-dominant stroma type (H&E x 100).

All core needle biopsy materials were scanned with a 5× objective. The area with the densest tumor stroma was determined, and a 10× objective was utilized for further examination. TSR was calculated as the ratio of the tumor stroma within the tumor mass to the total area covered by tumor cells and stroma. During the evaluation, necrosis, mucinous components, stroma surrounding in situ carcinoma, and benign parenchymal stroma were deliberately omitted from the analysis. The final assessment of TSR was conducted in regions where tumor cells were observed at the borders with a 10× objective^14,15^. TSR scores were ranked in 10% brackets, and patients with TSR<50% or equal were grouped as low TSR, while patients with TSR>50% were grouped as high TSR (Figure 1). TST was divided into three groups as C.D, F.D, and L.D according to the density of eosinophilic collagen in the stroma, immature collagen in the fibromyxoid background, and lymphocytic cell infiltration^11^.

The RCB score is given by considering the primary tumor bed, overall cancer cellularity, in situ cancer rate, lymph node metastasis, and the size of the metastatic area in the lymph node^16^. The Residual Cancer Burden Calculator system was used for RCB scoring (https://www3.mdanderson.org/app/medcalc/index.cfm?pagename=jsconvert3). Accordingly, the cases were divided into four different groups as RCB-0,1,2,3. For statistical analysis, according to the NAC response, cases with RCB0 and 1 were classified as the high NAC response group, while cases with RCB 2 and 3 were classified as the low NAC response group.

### Statistical analysis

Statistical analysis was carried out using the SPSS software (IBM, SPSS Inc., Version 25.0, Chicago, USA). Descriptive data related to categorical variables were expressed as frequencies and percentages (n, %). The association between categorical parameters was assessed by the chi-square test (Pearson chi-square and Fisher's exact test), considering the number of patient groups in the categories. To explore independent predictors for treatment response, potential prognostic factors ascertained by univariate analysis were further applied to multivariate logistic regression analysis, and an odds ratio (OR) with 95%CI was provided. An overall type-1 error level of 5% was utilized for statistical significance.

## RESULTS

### General clinicopathological features

All 158 patients included in the study were female, with an average age of 57 (30–90) years. The clinicopathological features of the cases are shown in Table 1. The average OS time of patients is 43 months, and the average DFS time is 28 months.

**Table 1:** Tumor-stroma type, tumor-stroma ratio, and associated clinicopathological parameters.

		Tumor-stroma type			p	Tumor-stroma ratio		p
		C.D	F.D	L.D		Low	High	
		n (%)	n (%)	n (%)		n (%)	n (%)	
Histological type	1	49 (86)	69 (86.3)	18 (85.7)	0.998	59 (89.4)	77 (83.7)	0.308
	2+3	8 (14)	11 (13.8)	3 (14.3)		7 (10.6)	15 (16.3)	
Nuclear grade	1+2	49 (86)	61 (76.3)	11 (52.4)	0.008	40 (60.6)	81 (88)	<0.001
	3	8 (14)	19 (23.8)	10 (47.6)		26 (39.4)	11 (12)	
Tumor stroma type	C.D					13 (19.7)	44 (47.8)	<0.001
	F.D					37 (56.1)	43 (46.7)	
	L.D					16 (24.2)	5 (5.4)	
Tumor stroma ratio	Low	13 (22.8)	37 (46.3)	16 (76.2)	<0.001			
	High	44 (77.2)	43 (53.8)	5 (23.8)				
Estrogen receptor	Negative	11 (19.3)	17 (21.3)	17 (81)	<0.001	31 (47)	14 (15.2)	<0.001
	Positive	46 (80.7)	63 (78.8)	4 (19)		35 (53)	78 (84.8)	
Progesterone receptor	Negative	18 (31.6)	24 (30)	17 (81)	<0.001	36 (54.5)	23 (25)	<0.001
	Positive	39 (68.4)	56 (70)	4 (19)		30 (45.5)	69 (75)	
Human epidermal growth factor receptor 2	0+1	31 (54.4)	43 (53.8)	10 (47.6)	0.979	25 (37.9)	59 (64.1)	0.003
	2	6 (10.5)	10 (12.5)	3 (14.3)		9 (13.6)	10 (10.9)	
	3	20 (35.1)	27 (33.8)	8 (38.1)		32 (48.5)	23 (25)	
Ki-67 proliferation index	0	9 (15.8)	18 (22.5)	0 (0)	0.049	4 (6.1)	23 (25)	0.002
	1	48 (84.2)	62 (77.5)	21 (100)		62 (93.9)	69 (75)	
Molecular subtype	LA	7 (12.3)	16 (20)	0 (0)	<0.001	3 (4.5)	20 (21.7)	<0.001
	LB	38 (66.7)	48 (60)	6 (28.6)		35 (53)	57 (62)	
	HER2	9 (15.8)	6 (7.5)	7 (33.3)		14 (21.2)	8 (8.7)	
	TN	3 (5.3)	10 (12.5)	8 (38.1)		14 (21.2)	7 (7.6)	
Angiolymphatic invasion	Negative	32 (56.1)	56 (70)	14 (66.7)	0.242	51 (77.3)	51 (55.4)	0.005
	Positive	25 (43.9)	24 (30)	7 (33.3)		15 (22.7)	41 (44.6)	
Lymph node metastasis	Negative	24 (42.1)	39 (48.8)	18 (85.7)	0.002	48 (72.7)	33 (35.9)	<0.001
	Positive	33 (57.9)	41 (51.3)	3 (14.3)		18 (27.3)	59 (64.1)	
RCB group (0+1 vs 2+3)	0+1	16 (28.1)	26 (32.5)	15 (71.4)	0.001	39 (59.1)	18 (19.6)	<0.001
	2+3	41 (71.9)	54 (67.5)	6 (28.6)		27 (40.9)	74 (80.4)	
Metastasis	Negative	46 (80.7)	66 (82.5)	17 (81)	0.961	56 (84.8)	73 (79.3)	0.378
	Positive	11 (19.3)	14 (17.5)	4 (19)		10 (15.2)	19 (20.7)	
EX status	Negative	51 (89.5)	73 (91.3)	18 (85.7)	0.75	61 (92.4)	81 (88)	0.368
	Positive	6 (10.5)	7 (8.8)	3 (14.3)		5 (7.6)	11 (12)	

C.D: collagen dominant; EX: death; F.D: fibromyxoid dominant; HER2: human epidermal growth factor receptor 2; LA: luminal A; LB: luminal B; L.D: lymphocytic dominant; RCB: Residual Cancer Burden; TN: triple-negative.

TST was C.D in 57 (36.1%) cases, F.D in 80 (50.6%) cases, and L.D in 21 (13.3%) cases. TSR was found to be low in 66 (41.8%) cases and high in 92 (58.2%) cases. The RCB score was 0 in 45 (28.5%) cases, 1 in 12 (7.5%) cases, 2 in 60 (38%) cases, and 3 in 41 (26%) cases.

### Clinicopathological parameters associated with tumor-stroma type and tumor-stroma ratio

Statistically, TST significantly correlated with TSR (p<0.001), nuclear grade (NG) (p=0.008), RCB score (p=0.001), molecular subtype (p<0.001), ER and PR expression (p<0.001), and Ki-67 proliferation index (p=0.049) (Table 1). While C.D type stroma was observed to be associated with low NG and low treatment response, L.D stroma was found to be correlated with high NG and high Ki-67 proliferation index.

Statistically TST (p<0.001), NG (p<0.001), ER and PR expression (p<0.001), HER2 expression (p=0.003), molecular subtype (p<0.001), and RCB score were observed to be associated with TSR (Table 1). Low TSR was found to be correlated especially with L.D stroma, high NG, TN, and HER2 molecular subtypes, and low RCB score.

### Clinicopathological parameters associated with residual cancer burden score

The RCB score demonstrated a statistically significant correlation with histological type, TST (p=0.018), TSR (p<0.001), molecular subtype (p<0.001), angiolymphatic invasion (p<0.001), lymph node metastasis (p<0.001), metastasis (p=0.001), and OS (p=0.038).

In the univariate analysis performed to determine the risk factors predicting the RCB score, histological type, TST, TSR, molecular subtype, lymph node metastasis, and angiolymphatic invasion were found to be associated with the RCB score. In multivariate analysis, molecular subtype [OR 7.040 (95%CI 2.311–21.446)] and TSR [OR 0.152 (95%CI 0.059–0.391)] were detected as independent risk factors for Residual Cancer Burden score (Table 2).

**Table 2:** Residual cancer burden score and logistic regression analysis.

	Univariate analysis				Multivariate analysis			
	P	OR	95%CI for OR		p	OR	95%CI for OR	
			Lower	Upper			Lower	Upper
Age	0.517	1.303	0.585	2.906				
Histological type (2+3 vs 1)	0.027	4.171	1.177	14.780	0.069	0.217	0.042	1.128
Nuclear grade	0.071	0.502	0.237	1.062	0.162	0.444	0.142	1.387
Pathologic stage (diameter) (pT2 vs pT1)	0.172	1.649	0.805	3.377	0.140	0.477	0.178	1.276
Pathologic stage (diameter) (pT3 vs pT1)	0.474	0.617	0.165	2.311	0.454	2.178	0.284	16.735
TST (F.D vs C.D)	0.580	0.811	0.385	1.705	0.569	1.322	0.506	3.454
TST (L.D vs C.D)	0.001	0.156	0.051	0.473	0.251	2.502	0.522	11.985
Tumor stroma ratio	<0.001	5.938	2.915	12.095	<0.001	0.152	0.059	0.391
Estrogen receptor	<0.001	8.665	3.961	18.956				
Progesterone receptor	<0.001	5.814	2.855	11.840				
HER2 (positive vs negative)	<0.001	0.123	0.057	0.268				
Ki-67 proliferation index	0.017	0.256	0.084	0.783				
Molecular subtype (HER2+TN vs LA+LB)	<0.001	0.113	0.051	0.251	0.001	7.040	2.311	21.446
Angiolymphatic invasion	<0.001	4.642	2.060	10.458	0.169	0.470	0.160	1.379
Perineural invasion	0.124	3.361	0.718	15.734	0.211	0.214	0.019	2.395
Lymph node metastasis	<0.001	5.631	2.755	11.510	0.313	0.598	0.221	1.622

Histological type, nuclear grade, pathologic stage (diameter), tumor-stroma type, tumor-stroma ratio group, molecular subtype, angiolymphatic invasion, perineural invasion, lymph node metastasis chemotherapy were selected as covariates. C.D: collagen dominant; F.D: fibromyxoid dominant; L.D: lymphocytic dominant; CI: confidence interval; OR: odds ratio; TST: tumor-stroma type; TN: triple-negative; LA: luminal A; LB: luminal B; HER2: human epidermal growth factor receptor 2.

## DISCUSSION

NAC is increasingly used in BC patients to avoid radical surgery and improve survival by reducing the size of the primary tumor^2,3^. However, in breast carcinomas, which are very heterogeneous tumors, the treatment response is not always found to be similar even in the same molecular subtypes. While a complete or almost complete pathological response was observed in some of the patients after NAC; some patients do not respond to NAC treatment.

Structural factors such as drug-metabolizing enzymes and transmembrane proteins are associated with treatment resistance^17^. Type 2 collagen may be the cause of drug resistance by structurally acting as a shield between the chemotherapeutic drug and the tumor cell^18^.

There is a consensus in studies that high tumor stroma is associated with poor prognosis in TN and HER2 molecular subtype BCs. However, there are different opinions about luminal-type BCs. Millar et al. reported high tumor stroma as a good prognostic factor in the luminal group^19^; however, Öztürk et al. reported it as a poor prognostic factor in the same group^20^.

In addition to the effects of tumor stroma on survival, a few studies also investigated its effect on NAC response. In the study of Mallya et al., which included 62 BC patients, high TSR was associated with poor NAC response^21^. In the study of Hagenaars et al., which included 375 HER2 negative BC patients, it was reported that low TSR was associated with a high MP score. This study revealed that the tumor stroma affects the NAC response more strongly in ER-negative patients than in ER-positive patients^22^.

In our study, we found that high TSR and C.D stroma were associated with NAC resistance, and TSR was found to be an independent risk factor in treatment response. Our data also show a high probability of response to NAC treatment in luminal molecular subtype BC patients with low TSR.

We believe that this outcome brings out the importance of questioning the requirement for NAC treatment, particularly in patients where the luminal molecular subtype is accompanied by high tumor stroma. Consequently, considering alternative treatments at an earlier stage becomes crucial for better outcomes. To reach a more definitive conclusion on this issue, results from different centers are needed.

It has been questioned whether small biopsies are sufficient for tumor stroma evaluation. A positive correlation was found between biopsy and resection in TSR scoring by Le et al.^23^. Since biopsy materials are the only available tissue samples for the assessment before NAC administration, in addition to their diagnostic importance, they should be used more effectively as predictive markers for treatment response.

## CONCLUSION

An effective risk analysis for NAC treatment is not always possible with current clinicopathological parameters. TSR and TST seem to be very useful in predicting NAC response as a practical marker that is reproducible and does not require additional cost and time.

For the TSR and TST to be included in the pathology reports of biopsy and resection materials, a consensus must be formed in both evaluation and scoring of them.
